# DNA methylation of *SFRP1*, *SFRP2*, and *WIF1* and prognosis of postoperative colorectal cancer patients

**DOI:** 10.1186/s12885-019-6436-0

**Published:** 2019-12-12

**Authors:** Xinyan Liu, Jinming Fu, Haoran Bi, Anqi Ge, Tingting Xia, Yupeng Liu, Hongru Sun, Dapeng Li, Yashuang Zhao

**Affiliations:** Department of Epidemiology, School of Public Health, Harbin Medical University, 157 Baojian Street, Nangang District, Harbin, 150086 Heilongjiang Province People’s Republic of China

**Keywords:** Colorectal cancer, Methylation, Prognosis, Wnt signaling pathway

## Abstract

**Background:**

As biomarkers, DNA methylation is used to detect colorectal cancer (CRC) and make assessment of CRC prognosis. The published findings showed the association between the methylation of *SFRP1*, *SFRP2*, and *WIF1*, located in the Wnt signaling pathway, and the prognosis of CRC were not consistent. Our study aimed to explore the potential possibility of *SFRP1*, *SFRP2*, and *WIF1* concomitant promoter methylation as prognostic biomarkers of postoperative CRC patients.

**Methods:**

As a total of 307 sporadic postoperative CRC patients were followed up, we detected *SFRP1*, *SFRP2*, and *WIF1* methylation obtained from tumor tissues and adjacent non-tumor tissues respectively on the basis of methylation-sensitive high resolution melting analysis. Univariate and multivariate Cox regressions were carried out so as to assess the potential possibility of *SFRP1*, *SFRP2*, and *WIF1* promoter methylation as predictors of prognosis. Confounders in our study were controlled by Propensity Score (PS) analysis.

**Results:**

The *SFRP1*, *SFRP2*, and *WIF1* methylation levels in tumor tissues were significantly higher than that in adjacent non-tumor tissues (*P* < 0.001). *SFRP2* hypermethylation was significantly associated with a favorable clinical outcome at the hazard ratio (HR) of 0.343 [95% confidence intervals (CI): 0.164–0.718, *P* = 0.005] and 0.410 (95% CI: 0.200–0.842, *P* = 0.015) in multivariate Cox regression and PS analysis, respectively. Co-hypermethylation of *SFRP1* and *SFRP2* was significantly associated with a favorable clinical outcome at the HR of 0.333 (95% CI: 0.159–0.694, *P* = 0.003) and 0.398 (95% CI: 0.192–0.821, *P* = 0.013) in multivariate Cox regression and PS analysis, respectively. Co-hypermethylation of *SFRP1*, *SFRP2* and *WIF1* was significantly associated with a favorable clinical outcome at the HR of 0.326 (95% CI: 0.117–0.908, *P* = 0.032) and 0.401 (95% CI: 0.146–1.106, *P* = 0.077) in multivariate Cox regression and PS analysis, respectively.

**Conclusions:**

*SFRP1*, *SFRP2*, and *WIF1* were frequently hypermethylated in CRC tumor tissues. It was apparent that the promoter hypermethylation of *SFRP2* and co-hypermethylation of *SFRP1* and *SFRP2* might be considered as independent prognostic predictors for survival advantage of postoperative CRC patients.

## Background

Colorectal cancer (CRC) has a high estimated death of 881,000 and ranks second in terms of mortality worldwide in 2018 [1]. The global burden of CRC is estimated to reach 1,100,000 cancer deaths by 2030 [2]. The 5-year relative survival rate for CRC patients is about 64.9%, which has remained less than 50% in low-income countries [3, 4]. Though removing the primary tumor by surgery is considered as the most common treatment for CRC patients, approximately 50% of postoperative patients will suffer a tumor recurrence over in the first three years [4]. So far, pathological staging and specific histological features have been seen as the most accurate prognostic predictors for postoperative CRC patients. However, patients with similar characteristics experience different prognosis. Therefore, more effective prognostic biomarkers might be a key to reduce deaths owing to CRC.

DNA methylation, as the most popular epigenetic alteration, could regulate gene expression through the modification of chromatin complexes and the recruitment of methyl-CpG domain-binding proteins around CpG islands [5]. Sufficiently powered clinical studies have revealed the potential feasibility of using specific methylated DNA signatures as CRC prognostic biomarkers in tumor tissues [6]. Studies have proposed that the hypermethylation of *CDKN2A* promoter and hypomethylation of *LINE-1* were independently associated with shorter survival in CRC patients [7, 8].

CRC results from an accumulation of genetic and epigenetic changes in intestinal epithelial cells. Nevertheless, the activation of the Wnt signaling pathway plays an essential role in the emergence of CRC. The DNA hypermethylation of *SFRP1*, *SFRP2*, and *WIF1*, which is located in the upstream of the canonical Wnt signaling pathway, leads to the downregulation of the gene expression, inhibition of gene function, activation of Wnt pathway and promotion of CRC [9, 10]. Also, the DNA hypermethylation of these genes could be used as a biomarker for detecting CRC [11–13]. *SFRP1* and *SFRP2* included in the *SFRPs* family, and *WIF1* are frequently hypermethylated in cell lines and tissues of CRC [9, 14]. This hypermethylation associated with a lack of expression could be restored by 5-aza-2′-deoxycytidine treatment [15–17]. Rawson et al. discovered that *SFRP1* methylation was not associated with recurrence-free survival in two large populations of CRC patients [18]. However, Kumar et al. found that promoter hypermethylation of *SFRP1* might be related to the poor prognosis of CRC [19]. Tang et al. revealed that promoter methylation of *SFRP2* in CRC tissues could be used as an independent prognostic factor for overall survival [20]. Samaei et al. reported that the overall survival rate of CRC patients with unmethylated *WIF1* was significantly higher than that with *WIF1* methylation by univariate analysis, whereas *SFRP2* methylation was not associated with overall survival rate [21]. The relationship between promoter methylation of *SFRP1*, *SFRP2*, and *WIF1* and the prognosis of CRC patients is unclear. Therefore, we would study and evaluate promoter methylation of *SFRP1*, *SFRP2*, and *WIF1* of the Wnt signaling pathway in CRC tissues. Furthermore, we also investigated the association between the methylation of these genes and the prognosis of postoperative CRC patients.

## Methods

### Study subjects and data collection

Three hundred and seven sporadic primary CRC patients confirmed by pathological diagnosis were collected from a follow-up study of 453 patients. These patients underwent surgical resection in the Third Affiliated Hospital of Harbin Medical University from November 2004 to July 2005 and from May 2007 to January 2008. And none of the patients had any other history of cancer or received pre-operative radiotherapy or chemotherapy. Clinical data of age, gender, tumor markers, clinic pathologic characteristics, and clinical information about disease and treatment were collected from the medical record registration system. All participants provided written informed consent. The Research Ethics Committee of Harbin Medical University approved this study.

The last follow-up date for this study was March 15, 2014 (which lasted 109 months). The time from patient’s surgery to death of various reasons or the last follow-up visit was defined as the overall survival (OS) time.

### DNA extraction and sodium bisulfate modification

Genomic DNA from patient’s tumor tissue and adjacent non-tumor tissue specimens was extracted by the classic phenol-chloroform method and then was stored at − 80 °C.

We used a commercially available DNA modification kit (EpiTect BisulfiteKit®, Qiagen, Hilden, Germany) to bisulfate the genomic DNA and stored them at − 20 °C. All processing steps were performed according to the instructions provided by the manufacturer.

### Methylation analysis of *SFRP1*, *SFRP2*, and *WIF1*

We detected and analyzed methylation of *SFRP1*, *SFRP2*, and *WIF1* using methylation-sensitive high resolution melting (MS-HRM) by LightCycler 480 (Roche Applied Science, Mannheim, Germany) with gene scanning software, as previously published [22].

All the target amplicons in our study were located in the promoter region of the three genes. The primers of *SFRP1* and *SFRP2* were designed as reported previously [23], and other primers were designed through Primer 5.0 software. All primers and conditions for the three genes in MS-HRM analysis were shown in Additional file 1: Table S1.

The whole reaction volume of PCR mixture was 5 μL consisting of 2.5 μL of 1 X Light-Cycler 480 High Resolution Melting Master Mix (Roche), 0.6 μL MgCl_2_ (3 mM), 0.125 μL of each forward and reverse primer (10 μM), 1.15 μL of polymerase chain reaction (PCR)-grade water and 0.5 μL of bisulfite-treated DNA respectively. The cycling protocol started with one cycle at 95 °C for 10 min, accompanied with 50 cycles at 95 °C for 10 s, a touchdown for 30 s (0.4 °C/step), 72 °C for 20 s, and a HRM step at 95 °C for 1 min, 40 °C for 1 min, and 70 °C for 5 s. The melting step strictly followed a continuous acquisition between 70 °C and 93 °C at 40 acquisitions per 1 °C.

A series of methylation standards were constructed, which included 100, 50, 35, 20, 10, 5 and 0% methylated DNA. In the context of universal unmethylated DNA, the series of standards were constructed by serially diluting the methylated control DNA into the unmethylated control according to mass concentration. A water-blank control was included in each batch and all samples were conducted in duplicate to ensure the repeatability of the experiment.

### Validation analysis with TCGA data

We further utilized The Cancer Genome Atlas (TCGA) datasets to validate the relationship between *SFRP1*, *SFRP2,* and *WIF1* methylation in tumor tissues and CRC patient prognosis. The DNA methylation detected by Illumina Human Methylation 450 in colon cancer and rectal cancer were downloaded and merged from UCSC Xena (https://xena.ucsc.edu/). The *cg04255616* probe and *cg25185173* probe were located in the target amplicon of *SFRP1* and *SFRP2* in our study, respectively, which were used to analyze DNA methylation and patient prognosis. However, none of the probes of *WIF1* deriving from the TCGA were located in the target amplicon of our study. Therefore, we used the average methylation values of all probes to replace the *WIF1* methylation level, and then analyzed DNA methylation and CRC patient prognosis (Additional file 2: Figure S1).

### Statistical analysis

The missing values of our research were filled by the multiple imputation method. We used the cut-off values of methylation, which were determined by the receiver operator characteristic (ROC) curve to clearly distinguish the tumor tissues from the adjacent non-tumor tissues and the hypomethylation from hypermethylation in tumor tissues. The χ2 test was used to assess the association between the methylation of *SFRP1*, *SFRP2*, and *WIF1* and clinic pathologic characteristics. We analyzed the survival rates using the life table method and compared the differences among the groups by log-rank test. The effects of *SFRP1*, *SFRP2*, and *WIF1* methylation on OS were estimated using univariate and multivariate Cox regression. Additionally, GraphPad Prism 7.0 was used to construct the survival curve. The statistical analyses were performed using SPSS version 23.0 software. Two-sided *P* values less than 0.05 were considered as significant.

Firstly, as *SFRP1* and *SFRP2* are members of the *SFRPs* family, we combined the two genes methylation as co-methylation-2 group to explore the association between gene methylation and CRC prognosis. Patients with promoter hypermethylation of *SFRP1* and *SFRP2* were classified as co-methylation-2H group, while others were as co-methylation-2 L group. Secondly, we combined the methylation of *SFRP1, SFRP2*, and *WIF1* co-methylation-3 group to explore the relationship between co-methylation and patient prognosis, since they regulate the Wnt signaling pathway to promote CRC development. On the other hand, patients with promoter hypermethylation of *SFRP1*, *SFRP2*, and *WIF1* were defined as co-methylation-3H group while others were as co-methylation-3 L group.

The propensity score (PS) method was used to balance the characteristics differences between the two methylation groups. Principally, a multivariate logistic regression model was established to estimate the PS, including the variables related to both gene methylation and CRC prognosis, or the CRC prognosis only. The PS based on it was defined as PS-1 [24]. In terms of the comprehensive literature, we set up PS-2 to evaluate all variables relevant to the prognosis of CRC patients for sensitivity analyses. The model incorporated the following factors such as age, gender, CEA [25], CA19–9 [26], multiple polyps [27], tumor location [28], TNM staging, pathological classification [29], histologic classification [30], differentiation degree, postoperative chemotherapy and postoperative radiotherapy.

Meanwhile, we also performed subgroup analyses based on age (< 45 years-old; ≥ 45 years-old), gender (male; female), tumor location (colon; rectum), TNM staging (I-II; III-IV) and postoperative chemotherapy as sensitivity analyses.

## Results

### *SFRP1*, *SFRP2*, and *WIF1* methylation in tumor and adjacent non-tumor tissues

We had detected the methylation of *SFRP1*, *SFRP2*, and *WIF1* for 187 adjacent non-tumor tissue specimens and 307 primary tumor tissue specimens. The *SFRP1*, *SFRP2*, and *WIF1* methylation levels in tumor tissues were significantly higher than that in adjacent non-tumor tissues (Mann-Whitney U test, *P* < 0.001) (Additional file 3: Table S2).

**Fig. 1:**
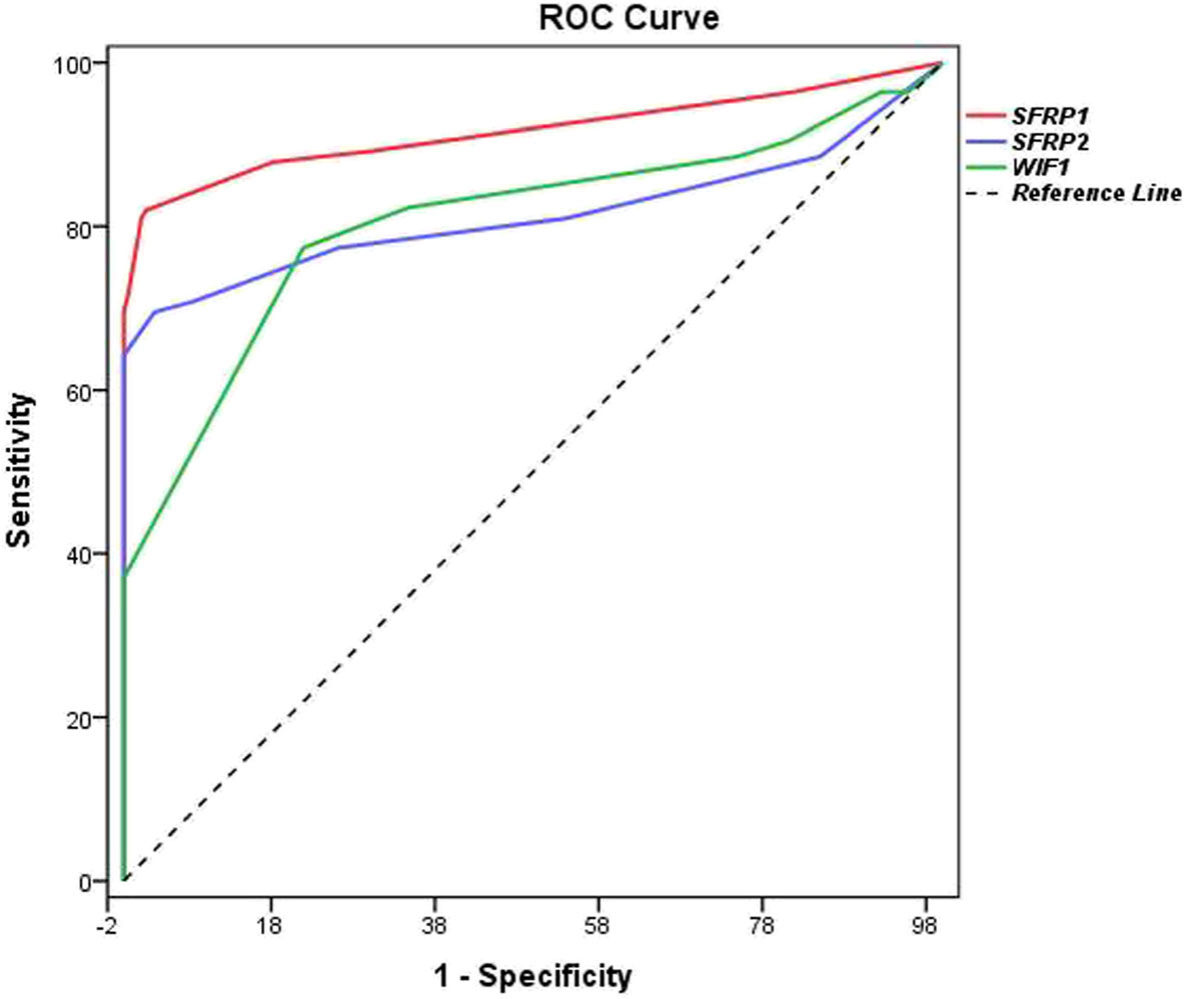
Receiver operator characteristic (ROC) curve of *SFRP1*, *SFRP2* and *WIF1* methylation from tumor tissues and non-tumor tissues

The cut-off values of *SFRP1*, *SFRP2*, and *WIF1* methylation were 10.0, 5.0 and 20.0%, respectively, which had high predictive ability to distinguish tumor tissues from adjacent non-tumor tissues. As shown in Fig 1, the area under curve (AUC) of *SFRP1*, *SFRP2* and *WIF1* were 0.916 (95% CI: 0.888–0.939), 0.814 (95% CI: 0.777–0.848) and 0.806 (95% CI: 0.768–0.840), respectively (Table 1). In adjacent non-tumor tissues, the number of patients with *SFRP1*, *SFRP2*, and *WIF1* methylation levels exceeding the cut-off value were 5 (2.7%), 7 (3.7%) and 41 (21.9%), respectively.

**Table 1:** The ROC analysis of gene methylation in tumor and adjacent non-tumor tissues

Gene	Cut-off value	Sensitivity	Specificity	AUC (95%CI) ^a^	*P* value
*SFRP1*	10.0%	82.0%	97.3%	0.916 (0.888–0.939)	< 0.0001
*SFRP2*	5.0%	69.6%	96.3%	0.814 (0.777–0.848)	< 0.0001
*WIF1*	20.0%	77.5%	78.1%	0.806 (0.768–0.840)	< 0.0001

^a^Area Under Curve (95% Confidence Interval)

For further survival analysis, the cut-off values of *SFRP1*, *SFRP2*, and *WIF1* methylation for distinguishing the survival status accounted for 10.0, 50.0, and 50.0% in tumor tissues. According to the cut-off values, the patients were categorized into hypomethylation group and hypermethylation group.

### The association between *SFRP1*, *SFRP2*, and *WIF1* methylation in tumor tissues and clinic pathologic characteristics of CRC patients

The median age of diagnosis for 307 CRC patients was 58 years old (varying from 25 to 80 years old) while the male-to-female ratio was 1.42. Promoter methylation of *SFRP1* was associated with age (*P* = 0.040), lymph nodes involved (*P* = 0.036), histologic classification (*P* = 0.044) and differentiation degree (*P* = 0.011). *SFRP2* promoter methylation was associated with TNM staging (*P* = 0.042), and the proportion of patients with hypermethylation was higher in the I-II stage. *WIF1* promoter methylation was associated with pathological classification (*P* = 0.023) (Table 2).

**Table 2:** Association of promoter methylation with clinic pathologic characteristics of CRC patients (*N* = 307)

Characteristics	Total	*SFRP1* N (%)			*SFRP2* N (%)			*WIF1* N (%)		
	N	Hypo-M ^a^	Hyper-M ^b^	*P* value	Hypo-M	Hyper-M	*P* value	Hypo-M	Hyper-M	*P* value
All cases	307	55 (17.9)	252 (82.1)		264 (86.0)	43 (14.0)		224 (73.3)	83 (26.7)	
Age				0.040			0.382			0.459
< 45 years-old	33	7 (12.7)	26 (10.3)		26 (9.8)	7 (16.3)		27 (12.1)	6 (7.2)	
45~60 years-old	132	31 (56.4)	101 (40.1)		113 (42.8)	19 (44.2)		96 (42.9)	36 (43.4)	
≥ 60 years-old	142	17 (30.9)	125 (49.6)		125 (47.4)	17 (39.5)		101 (45.0)	41 (49.4)	
Gender				0.766			0.944			0.224
Male	180	33 (60.0)	147 (58.3)		155 (58.7)	25 (58.1)		136 (60.7)	44 (53.0)	
Female	127	22 (40.0)	105 (41.7)		109 (41.4)	18 (41.9)		88 (39.3)	39 (47.0)	
CEA				0.930			0.207			0.824
< 5 ng/mL	130	23 (41.8)	107 (42.5)		108 (40.9)	22 (51.2)		94 (42.0)	36 (43.4)	
≥ 5 ng/mL	177	32 (58.2)	145 (57.5)		156 (59.1)	21 (48.8)		130 (58.0)	47 (56.6)	
CA19–9				0.114			0.180			0.327
< 37 U/mL	232	37 (67.3)	195 (77.4)		196 (74.2)	36 (83.7)		166 (74.1)	66 (79.5)	
≥ 37 U/mL	75	18 (32.7)	57 (22.6)		68 (25.8)	7 (16.3)		58 (25.9)	17 (20.5)	
Multiple polyps				0.600			0.127			0.115
No	220	41 (74.5)	179 (71.0)		185 (70.1)	35 (81.4)		155 (69.2)	65 (78.3)	
Yes	87	14 (25.5)	73 (29.0)		79 (29.9)	8 (18.6)		69 (30.8)	18 (21.7)	
Tumor location				0.424			0.326			0.809
Colon	115	18 (32.7)	97 (38.5)		96 (36.4)	19 (44.2)		83 (37.1)	32 (38.6)	
Rectum	192	37 (67.3)	155 (61.5)		168 (63.6)	24 (55.8)		141 (62.9)	51 (61.4)	
TNM Staging				0.121			0.042			0.311
I- II	163	24 (43.6)	139 (55.2)		134 (50.8)	29 (67.4)		115 (51.3)	48 (57.8)	
III-IV	144	31 (56.4)	113 (44.8)		130 (49.2)	14 (32.6)		109 (48.7)	35 (42.2)	
Tumor invasion				0.364			0.111			0.183
T1- T3	151	24 (42.9)	127 (50.4)		125 (47.3)	26 (60.5)		105 (46.9)	46 (55.4)	
T4	156	31 (56.4)	125 (49.6)		139 (52.7)	17 (39.5)		119 (53.1)	37 (44.6)	
Lymph nodes involved				0.036			0.056			0.403
N0	173	24 (43.6)	149 (59.1)		143 (54.2)	30 (69.8)		123 (54.9)	50 (60.2)	
N1- N2	134	31 (56.4)	103 (40.9)		121 (45.8)	13 (30.2)		101 (45.1)	33 (39.8)	
Metastasis status				0.231			0.165			0.384
M0	284	53 (96.4)	231 (91.7)		242 (91.7)	42 (97.7)		209 (93.3)	75 (90.4)	
M1	23	2 (3.6)	21 (8.3)		22 (8.3)	1 (2.3)		15 (6.7)	8 (9.6)	
Pathological classification				0.053			0.316			0.023
Prominence	199	31 (56.4)	168 (66.7)		168 (63.6)	31 (72.1)		135 (60.3)	64 (77.1)	
Ulceration	86	16 (29.1)	70 (27.8)		78 (29.5)	8 (18.6)		71 (31.7)	15 (18.1)	
Others	22	8 (14.5)	14 (5.5)		18 (6.9)	4 (9.3)		18 (8.0)	4 (4.8)	
Histologic classification				0.044			0.113			0.361
Adenocarcinoma	235	35 (63.6)	200 (79.4)		203 (76.9)	32 (74.4)		176 (78.6)	59 (71.1)	
Mucinous adenocarcinoma	68	19 (34.5)	49 (19.4)		59 (22.3)	9 (20.9)		45 (20.1)	23 (27.7)	
Others	4	1 (1.9)	3 (1.2)		2 (0.8)	2 (4.7)		3 (1.3)	1 (1.2)	
Differentiation degree				0.011			0.083			0.255
Poor	49	15 (27.3)	34 (13.5)		46 (17.4)	3 (7.0)		39 (17.4)	10 (12.0)	
Moderate or well	258	40 (72.7)	218 (86.5)		218 (82.6)	40 (93.0)		185 (82.6)	73 (88.0)	

^a^Hypomethylation

^b^Hypermethylation

### The association between *SFRP1*, *SFRP2*, and *WIF1* methylation in tumor tissues and CRC prognosis

One hundred and nine months’ follow-up revealed 41.4% (127/307) of the CRC patients died while 46.9% (144/307) alive with the follow-up mean of 76.90 months and 73-month median of OS time for all patients (Additional file 4: Table S3).

The 5-year and 8-year survival rates of patients with *SFRP1* hypermethylation were 68.3 and 56.2%, respectively, which were significantly higher than that of patients with hypomethylation (47.2 and 25.8%, respectively). The 3-year, 5-year and 8-year survival rate of patients with *SFRP2* hypermethylation were 92.8, 90.4, and 82.2%, respectively, which were significantly higher than that of patients with hypomethylation (72.0, 60.3 and 45.5%, respectively) (Table 3).

**Table 3:** The overall survival rates at 1, 3, 5 and 8 year in groups stratified by methylation in tumor tissues (*N* = 307)

Groups	1 year		3 year		5 year		8 year	
	OSR (SE) ^a^	*P* value	OSR (SE)	*P* value	OSR (SE)	*P* value	OSR (SE)	*P* value
All patients (*N* = 307)	0.915 (0.018)		0.748 (0.027)		0.646 (0.028)		0.518 (0.039)	
*SFRP1*		0.229		0.071		0.005		0.003
Hypomethylation (*N* = 55)	0.872 (0.052)		0.660 (0.070)		0.472 (0.071)		0.258 (0.116)	
Hypermethylation (*N* = 252)	0.925 (0.019)		0.767 (0.030)		0.683 (0.031)		0.562 (0.042)	
*SFRP2*		0.205		0.015		0.001		0.000
Hypomethylation (*N* = 246)	0.905 (0.021)		0.720 (0.031)		0.603 (0.032)		0.455 (0.048)	
Hypermethylation (*N* = 43)	0.976 (0.023)		0.928 (0.040)		0.904 (0.046)		0.822 (0.062)	
*WIF1*		0.044		0.477		0.793		0.598
Hypomethylation (*N* = 224)	0.932 (0.020)		0.755 (0.032)		0.627 (0.034)		0.491 (0.046)	
Hypermethylation (*N* = 83)	0.868 (0.041)		0.730 (0.055)		0.700 (0.051)		0.595 (0.073)	
Co-methylation-2		0.205		0.015		0.001		0.000
Co-methylation-2 L ^b^	0.905 (0.020)		0.720 (0.031)		0.603 (0.032)		0.462 (0.044)	
Co-methylation-2H ^c^	0.976 (0.023)		0.928 (0.040)		0.904 (0.046)		0.822 (0.062)	
Co-methylation-3		0.624		0.111		0.013		0.004
Co-methylation-3 L ^d^	0.912 (0.019)		0.735 (0.029)		0.623 (0.030)		0.493 (0.039)	
Co-methylation-3H ^e^	0.957 (0.043)		0.913 (0.059)		0.913 (0.059)		0.852 (0.080)	

^a^Overall Survival Rate (Standard Error)

^b^Co-methylation-2 L: patients with promoter hypomethylation of at least one gene (*SFRP1* or *SFRP2*)

^c^Co-methylation-2H: patients with promoter hypermethylation of *SFRP1* and *SFRP2*

^d^Co-methylation-3 L: patients with promoter hypomethylation of at least one gene (*SFRP1* or *SFRP2* or *WIF1*)

^e^Co-methylation-3H: patients with promoter hypermethylation of *SFRP1*, *SFRP2* and *WIF1*

In the multivariate Cox regression, the results showed that CA19–9, TNM staging, differentiation degree and postoperative radiotherapy were independent prognostic biomarkers for CRC patients (Additional file 5: Table S4).

**Fig. 2:**
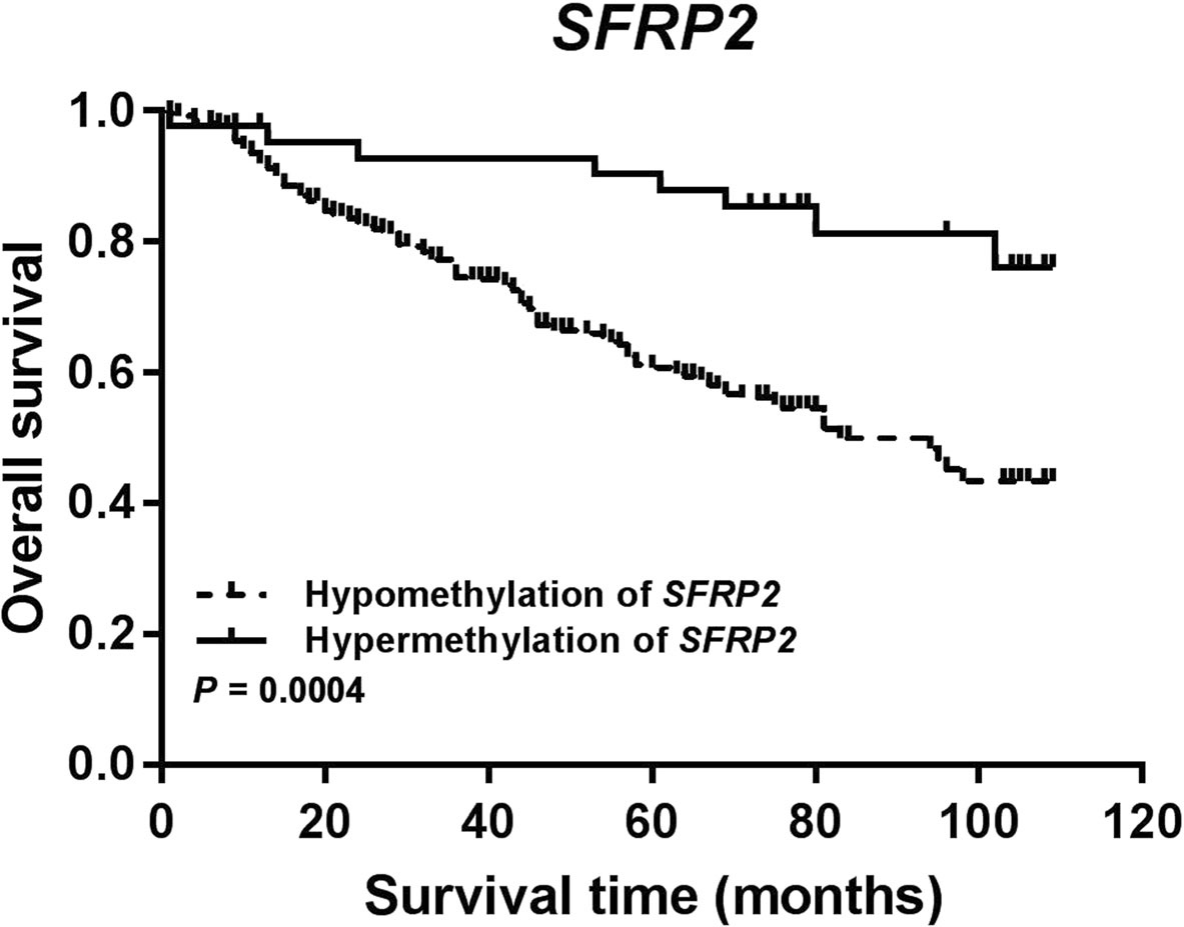
Survival curve of CRC patients with *SFRP2* hypermethylation and *SFRP2* hypomethylation

**Fig. 3:**
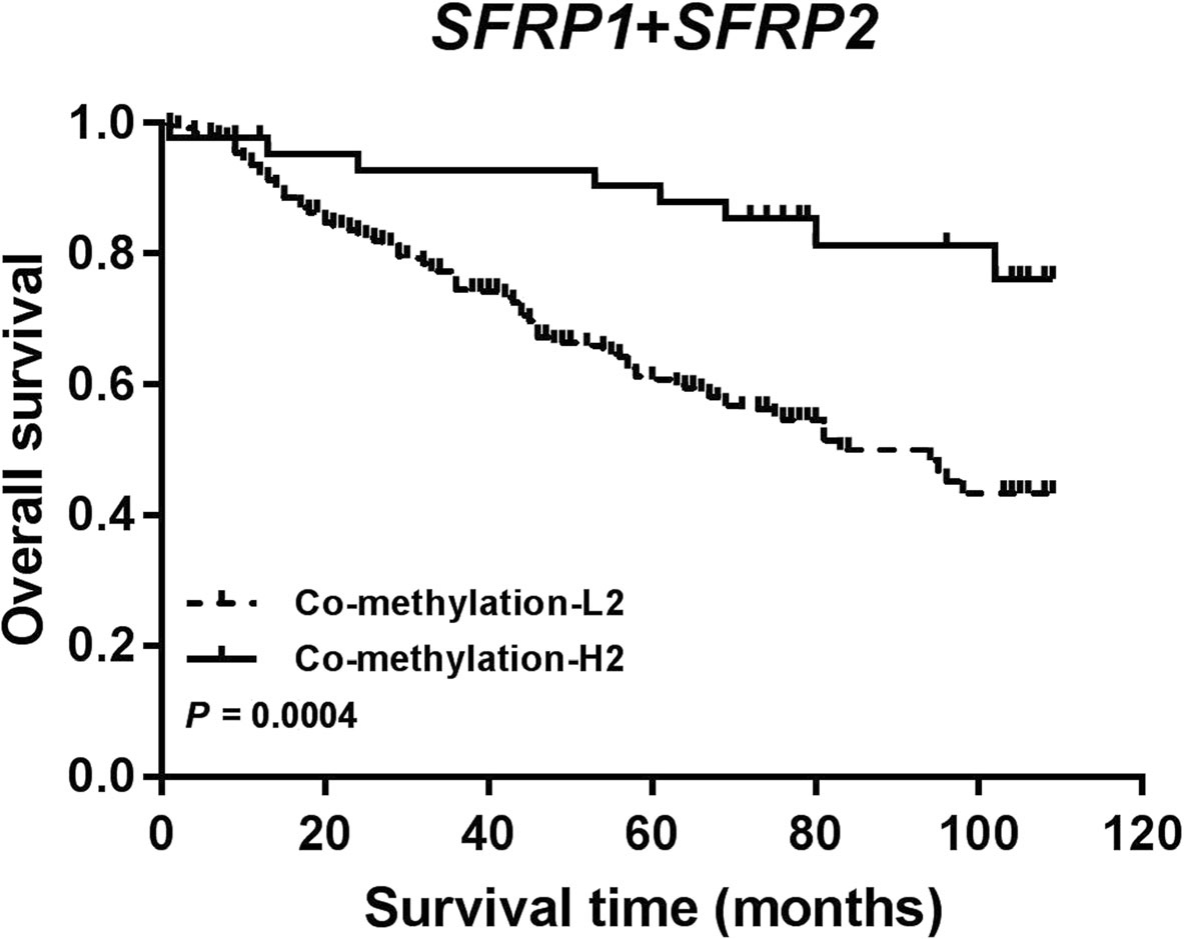
Survival curve of CRC patients with co-methylation-H2 and co-methylation-L2

*SFRP2* hypermethylation was significantly associated with a favorable clinical outcome with the HR of 0.343 (95% CI: 0.164–0.718, *P* = 0.005), 0.410 (95% CI: 0.200–0.842, *P* = 0.015) and 0.455 (95% CI: 0.219–0.944, *P* = 0.034) in multivariate Cox regression, PS-1 and PS-2 analysis, respectively (Table 4, Fig. 2). Co-hypermethylation of *SFRP1* and *SFRP2* was significantly associated with a favorable clinical outcome with the HR of 0.333 (95% CI: 0.159–0.694, *P* = 0.003), 0.398 (95%CI: 0.192–0.821, *P* = 0.013) and 0.442 (95% CI: 0.212–0.923, *P* = 0.030) in multivariate Cox regression, PS-1 and PS-2 analysis, respectively (Table 4, Fig. 3). Co-hypermethylation of *SFRP1*, *SFRP2*, and *WIF1* was significantly associated with a favorable clinical outcome with the HR of 0.326 (95%CI: 0.117–0.908, *P* = 0.032) in multivariate Cox regression, however, the results showed that this co-methylation was not associated with prognosis in PS-1 analysis, with HR of 0.401 (95% CI: 0.146–1.106, *P* = 0.077) (Table 4).

**Table 4:** Univariate and multivariate Cox analysis for association between methylation and OS in 307 CRC patients

Variables	Number		Univariate Cox		Multivariate Cox		Propensity score-1		Propensity score-2	
	Patients (*N* = 307)	Deaths (*N* = 127)	Crude HR (95% CI)	*P* value	Adjusted HR ^a^ (95% CI)	*P* value	Adjusted HR ^a^ (95% CI)	*P* value	Adjusted HR ^b^ (95% CI)	*P* value
*SFRP1*				0.001		0.070		0.084		0.096
Hypomethylation	55	31	1.000		1.000		1.000		1.000	
Hypermethylation	252	96	0.505 (0.336–0.760)		0.672 (0.437–1.032)		0.686 (0.447–1.051)		0.688 (0.443–1.069)	
*SFRP2*				0.001		0.005		0.015		0.034
Hypomethylation	264	119	1.000		1.000		1.000		1.000	
Hypermethylation	43	8	0.307 (0.149–0.633)		0.343 (0.164–0.718)		0.410 (0.200–0.842)		0.455 (0.219–0.944)	
*WIF1*				0.399		0.688		–		–
Hypomethylation	224	98	1.000		1.000		–		–	
Hypermethylation	83	29	0.836 (0.552–1.267)		1.092 (0.710–1.682)		–		–	
Co-methylation-2				0.001		0.003		0.013		0.030
Co-methylation-2 L ^c^	264	119	1.000		1.000		1.000		1.000	
Co-methylation-2H ^d^	43	8	0.298 (0.145–0.612)		0.333 (0.159–0.694)		0.398 (0.192–0.821)		0.442 (0.212–0.923)	
Co-methylation-3				0.018		0.032		0.077		0.155
Co-methylation-3 L ^e^	284	123	1.000		1.000		1.000		1.000	
Co-methylation-3H ^f^	23	4	0.300 (0.110–0.814)		0.326 (0.117–0.908)		0.401 (0.146–1.106)		0.479 (0.174–1.321)	

^a^Controlling for the variables which included age, gender, CEA, CA19–9, TNM staging, pathological classification, differentiation degree and postoperative radiotherapy

^b^Controlling for the variables which included age, gender, CEA, CA19–9, multiple polyps, tumor location, TNM staging, pathological classification, histologic classification, differentiation degree, postoperative chemotherapy and postoperative radiotherapy

^c^Co-methylation-2 L: patients with promoter hypomethylation of at least one gene (*SFRP1* or *SFRP2*)

^d^Co-methylation-2H: patients with promoter hypermethylation of *SFRP1* and *SFRP2*

^e^Co-methylation-3 L: patients with promoter hypomethylation of at least one gene (*SFRP1* or *SFRP2* or *WIF1*)

^f^Co-methylation-3H: patients with promoter hypermethylation of *SFRP1*, *SFRP2* and *WIF1*

For colon cancer patients, *SFRP2* hypermethylation patients and co-hypermethylation of *SFRP1* and *SFRP2* patients had a significantly favorable outcome through multivariate Cox regression, PS-1 and PS-2 analysis. For male CRC patients, *SFRP2* hypermethylation patients and co-hypermethylation of *SFRP1* and *SFRP2* patients had a considerably positive outcome only in multivariate Cox regression. In CRC patients with TNM staging III/IV, *SFRP2* hypermethylation patients had a significantly definite outcome with the HR of 0.280 (95% CI: 0.097–0.809, *P* = 0.019) in multivariate Cox regression. Co-hypermethylation of *SFRP1* and *SFRP2* patients had significantly favorable outcome with the HR of 0.263 (95% CI: 0.092–0.751, *P* = 0.013) and 0.352 (95% CI: 0.127–0.970, *P* = 0.044) in multivariate Cox regression and PS-1, respectively. For postoperative chemotherapy patients, co-hypermethylation of *SFRP1* and *SFRP2* patients had a relatively optimistic outcome in multivariate Cox regression (Table 5).

**Table 5:** Subgroup analysis on the association between methylation and OS ^a^

Symbol	Subgroup	Univariate Cox		Multivariate Cox		Propensity score-1		Propensity score-2	
		Crude HR (95%CI)	*P* value	Adjusted HR ^b^ (95%CI)	*P* value	Adjusted HR ^b^ (95%CI)	*P* value	Adjusted HR ^c^ (95%CI)	*P* value
*SFRP2*	Age								
	< 45 years-old	0.150 (0.020–1.148)	0.068	0.021 (0.001–0.348)	0.007	0.126 (0.016–1.025)	0.053	0.161 (0.021–1.263)	0.082
	≥ 45 years-old	0.346 (0.160–0.748)	0.007	0.414 (0.188–0.911)	0.028	0.479 (0.221–1.035)	0.061	0.537 (0.246–1.173)	0.119
	Gender								
	Male	0.373 (0.149–0.935)	0.035	0.357 (0.135–0.945)	0.038	0.471 (0.186–1.197)	0.113	0.551 (0.213–1.427)	0.219
	Female	0.214 (0.066–0.698)	0.011	0.311 (0.091–1.056)	0.061	0.328 (0.098–1.102)	0.071	0.291 (0.085–1.005)	0.051
	Tumor location								
	Colon	0.177 (0.043–0.733)	0.017	0.120 (0.027–0.527)	0.005	0.203 (0.049–0.843)	0.028	0.215 (0.052–0.899)	0.035
	Rectum	0.413 (0.178–0.960)	0.040	0.570 (0.237–1.366)	0.207	0.673 (0.291–1.557)	0.355	0.722 (0.308–1.695)	0.455
	TNM Staging								
	I-II	0.372 (0.132–1.048)	0.061	0.375 (0.126–1.119)	0.079	0.442 (0.156–1.254)	0.125	0.428 (0.150–1.221)	0.112
	III-IV	0.311 (0.113–0.859)	0.024	0.280 (0.097–0.809)	0.019	0.374 (0.133–1.048)	0.061	0.452 (0.160–1.272)	0.132
	Postoperative chemotherapy								
	No	0.249 (0.078–0.797)	0.019	0.284 (0.085–0.945)	0.040	0.381 (0.117–1.239)	0.109	0.365 (0.111–1.203)	0.098
	Yes	0.360 (0.142–0.910)	0.031	0.392 (0.150–1.026)	0.056	0.448 (0.177–1.130)	0.089	0.535 (0.207–1.384)	0.197
*SFRP1+ SFRP2*	Age								
	< 45 years-old	0.150 (0.020–1.148)	0.068	0.021 (0.001–0.348)	0.007	0.119 (0.014–1.025)	0.053	0.151 (0.019–1.225)	0.077
	≥ 45 years-old	0.334 (0.155–0.720)	0.005	0.399 (0.181–0.879)	0.023	0.463 (0.212–1.008)	0.052	0.523 (0.238–1.152)	0.108
	Gender								
	Male	0.356 (0.143–0.886)	0.026	0.339 (0.130–0.884)	0.027	0.448 (0.179–1.119)	0.086	0.526 (0.206–1.343)	0.179
	Female	0.214 (0.066–0.698)	0.011	0.311 (0.091–1.056)	0.061	0.328 (0.098–1.103)	0.072	0.304 (0.090–1.022)	0.054
	Tumor location								
	Colon	0.177 (0.043–0.733)	0.017	0.120 (0.027–0.527)	0.005	0.200 (0.048–0.832)	0.027	0.213 (0.051–0.890)	0.034
	Rectum	0.397 (0.172–0.918)	0.031	0.546 (0.227–1.312)	0.176	0.656 (0.278–1.551)	0.337	0.708 (0.297–1.690)	0.437
	TNM Staging								
	I-II	0.372 (0.132–1.048)	0.061	0.375 (0.126–1.119)	0.079	0.445 (0.157–1.260)	0.127	0.434 (0.152–1.237)	0.118
	III-IV	0.295 (0.108–0.809)	0.018	0.263 (0.092–0.751)	0.013	0.352 (0.127–0.970)	0.044	0.419 (0.147–1.193)	0.103
	Postoperative chemotherapy								
	No	0.249 (0.078–0.797)	0.019	0.284 (0.085–0.945)	0.040	0.381 (0.117–1.239)	0.109	0.360 (0.109–1.191)	0.094
	Yes	0.343 (0.137–0.861)	0.023	0.372 (0.144–0.961)	0.041	0.426 (0.168–1.076)	0.071	0.517 (0.200–1.332)	0.172

^a^All HR values were referenced by hypomethylation

^b^Controlling for the variables which included age, gender, CEA, CA19–9, TNM staging, pathological classification, differentiation degree and postoperative radiotherapy

^c^Controlling for the variables which included age, gender, CEA, CA19–9, multiple polyps, tumor location, TNM staging, pathological classification, histologic classification, differentiation degree, postoperative chemotherapy and postoperative radiotherapy

### Validation results with TCGA data

The TCGA dataset included a total of 399 patients, of which 88 died. The follow-up period ranged from 6 to 4502 days. The median age of diagnosis was 66 years old (ranging from 31 to 90 years old), and the male-to-female ratio was 1.17.

The *cg04255616* probe (*SFRP1*), *cg25185173* probe (*SFRP2*), and *WIF1* methylation levels of adjacent non-tumor tissues were significantly lower than those of tumor tissues (Mann-Whitney U test, *P* < 0.001). There was no direct relevance between the methylation of *cg04255616* probe (*SFRP1*), *cg25185173* probe (*SFRP2*) and the prognosis of CRC patients in multivariate Cox regression. *WIF1* hypomethylation was significantly associated with survival advantage in CRC patients, with the HR of 2.022 (95%CI: 1.309–3.124, *P* = 0.002) in multivariate Cox regression. In addition, the co-methylation of *SFRP1* and *SFRP2* and the co-methylation of *SFRP1*, *SFRP2*, and *WIF1* were not significantly associated with the prognosis of CRC (Additional file 6: Table S5).

## Discussion

The Wnt signaling pathway plays an essential role in the development and progression of CRC. Promoter hypermethylation of *SFRP1*, *SFRP2*, and *WIF1* involved in CRC has been described as negative regulators of the canonical Wnt pathway. Evidence has shown that DNA methylation could be developed as prognostic biomarkers in CRC [6]. However, the implications of *SFRP1*, *SFRP2*, and *WIF1* promoter methylation on the prognosis of CRC patients were not clear. As far as we know, it is the first study on investigating the association between *SFRP1*, *SFRP2*, and *WIF1* concomitant promoter methylation and prognosis of CRC patients.

MS-HRM is a simple, reliable and high sensitive technique, which can even assess individual CpG site and detect low-abundance (as low as 0.1–1%) methylation [22]. Liu et al. [31] had indicated significant consistency of gene methylation between the detection of pyrosequencing methods and MS-HRM in our laboratory.

In this study, we found that the promoter methylation level of *SFRP1*, *SFRP2*, and *WIF1* was enormously higher in tumor tissues than that in adjacent non-tumor tissues. The findings were similar to those of previously published studies [17, 32, 33]. The sensitivity and specificity of *SFRP1* were 82.0 and 97.3% respectively for methylation in tumor tissues and adjacent non-tumor tissues in our study. Zhang et al. showed that the sensitivity and specificity of *SFRP1* were 89 and 86% respectively for the methylation detected in stool DNA [34]. Due to different methylation detection methods and test samples, it might explain why results difference between us and other researchers exist. The improved specificity would increase the positive predictive value in judging CRC tumor tissue and adjacent non-tumor tissue.

*SFRP1* hypermethylation tended to occur frequently to tumors of patients with ≥60 years old, no lymph nodes involved, adenocarcinoma and moderate or well differentiation degree in the current study. Hu et al. also revealed that the percentage of methylated reference was higher in patients at more than 60 and no lymph nodes metastasis. However, there is no radical difference in methylation between the different patients [9].. Galamb et al. also proposed that hypermethylation of the *SFRP1* promoter was associated with aging [35]. Kumar et al. reported that *SFRP1* promoter methylation was associated with lymph nodes metastasis [19]. Bartak discovered that the *SFRP1* methylation was not connected with lymph node metastasis [36]. We found that *SFRP2* methylation was associated with TNM staging while *WIF1* methylation with pathological classification. Other researches did not report similar results [21, 36, 37]. The different findings above might be determined by different methods of methylation detections, sample sizes of the study cohort or compositions of the sample.

We found that *SFRP2* methylation had a more significant impact on prognosis, with the HR of 0.343 (0.164–0.718) in multivariate Cox regression. In addition, patients with hypermethylation of both *SFRP1* and *SFRP2* and patients with hypermethylation of *SFRP1*, *SFRP2*, and *WIF1* were at lower risk of death than that with non-all hypermethylation. However, the relationship between co-methylation-3 (*SFRP1*, *SFRP2*, and *WIF1*) and the prognosis was inconsistent with multivariate Cox regression and PS-1, with the HR of 0.401 (0.146–1.106) in PS-1. Therefore, further research is needed to validate this result. Combining these results, we believed that the co-methylation of multiple genes was better in evaluating the prognosis of patients compared with single genes.

PS is considered as a powerful method for balancing numbers of confounding factors in observational studies [38]. After PS adjustment, the relationships between *SFRP2* methylation, co-methylation-2 and the CRC prognosis were slightly increased compared with that based on the crude HR, which also suggested the reliability of the results. The PS-1 model focused on age, gender, CEA, CA19–9, TNM staging, pathological classification, differentiation degree and postoperative radiotherapy while the PS-2 model concentrated on PS-1 and the factors of multiple polyps, tumor location, histologic classification and postoperative chemotherapy. Our findings stem from objective analysis instead of external confounding factors.

Usually, as tumor-suppressor gene, *SFRP1* and *SFRP2* methylation were inversely correlated with the mRNA expression, and the expressions were increased after demethylation treatment in CRC cell lines [9]. Our results were contradicted with the above hypothesis that silencing of *SFRP1* and *SFRP2* by hypermethylation caused a better prognosis for the CRC patients. Furthermore, other researchers had the same conclusion as ours. Perez et al. found that *RASSF2* hypermethylation was associated with a better prognosis of breast cancer [39]. Therefore, we believed that the gene methylation might lead to additional genetic changes or interact with other factors, rather than dependent on gene methylation alone.

Our subgroup analyses were stratified by age, gender, tumor location, TNM staging and postoperative chemotherapy. First, our result showed that patients with *SFRP2* hypermethylation or co-hypermethylation of *SFRP1* and *SFRP2* had a lower risk of death in groups of ≥45 years old, male, colon cancer and TNM staging III-IV in traditional univariate and multivariate Cox. In addition, the patients with *SFRP2* hypermethylation or co-hypermethylation of *SFRP1* and *SFRP2*, who did not receive postoperative chemotherapy, had a lower risk of death while their HRs was consistent. So, we hypothesized that *SFRP2* methylation played a more significant impact than *SFRP1* on prognosis. The postoperative chemotherapy with co-hypermethylation of *SFRP1* and *SFRP2* was positively associated with patients’ prognosis, whereas *SFRP2* methylation was negatively associated with patients’ prognosis. Therefore, we hypothesize that *SFRP1* methylation and *SFRP2* methylation synergistically affect patients’ outcomes. PS has been proved to be a useful, innovative and creative statistical method for evaluating intervention effects in non-experimental or observational studies. PS analysis confirmed the stability and reliability of the results of multivariate survival analysis. Through propensity score analysis, we found that co-hypermethylation of *SFRP1* and *SFRP2* could be more suitable for prognostic risk assessment of CRC than other predictors. Furthermore, the multiple combinations of genes with similar functions and structures could increase the clinical evaluation value of methylation. Secondly, it is well known that CRC patients with TNM staging III-IV have a poor prognosis. Nevertheless, we found that subgroups of promoter hypermethylation of *SFRP2* or co-hypermethylation of *SFRP1* and *SFRP2* had a better prognosis than those of hypomethylation among patients with TNM staging III-IV. The findings triggered us to put them onto the clinical practice and precisely assess the individualized treatment of CRC patients.

With the aid of similar statistical analysis, we deeply explored the relationship between the methylation of all cg sites of the three genes and the prognosis in the TCGA database (Additional file 7: Table S6). There were no statistically significant association between the methylation of *cg04255616* probe (*SFRP1*) and *cg25185173* probe (*SFRP2*) and prognosis of CRC patients while the *WIF1* methylation was significantly associated with CRC prognosis. Unlike the Caucasian and African America as the primary research objects of the TCGA database, all participants in our research were Chinese. In addition, two probes in the TCGA database were located in the promoter region of *SFRP1* and *SFRP2*. The amplicons with multiple CpG sites in the promoter regions contained precise representativeness. Similarly, we applied the average methylation values of all probes in the TCGA as the *WIF1* methylation level for prognostic analysis. The number of cg sites (probes) in the TCGA is much more than that in the target amplicon of our study, as might cause different results. Interestingly, Chen et al. found that hypermethylation of *NDRG4* promoter was a predictor of poor overall survival in gastric cancer in China. However, the opposite results were observed in the TCGA cohort. They also believed that racial differences of study population caused different outcomes [40].

Up to now, this is a novel study about the methylation of Wnt signaling pathway related to genes on the prognosis of postoperative CRC patients. By MS-HRM, we had examined tumor tissues and adjacent non-tumor tissues obtained from surgical patients. Compared to other forms of clinical samples, tissue samples containing a large number of cells were used for the detection of gene methylation. However, some limitations should also be considered. Firstly, our study did not involve the assessment of tumor-specific death. Secondly, due to limited collected information about the treatment of CRC patients, the analyses of the associations between methylation of *SFRP1*, *SFRP2*, and *WIF1* and treatment decision were restricted to some extent, which might be used to establish more personalized treatment strategies.

## Conclusions

The promoter of *SFRP1*, *SFRP2*, and *WIF1* were frequently hypermethylated in CRC tumor tissues. Promoter hypermethylation of *SFRP2,* co-hypermethylation of *SFRP1* and *SFRP2*, and co-hypermethylation of *SFRP1*, *SFRP2*, and *WIF1* could be considered as independent prognostic predictors for the survival advantage in patients with CRC. For colon cancer, patients with promoter hypermethylation of *SFRP1* or co-hypermethylation of *SFRP1* and *SFRP2* had higher overall survival. In TNM staging III-IV, patients with co-hypermethylation of *SFRP1* and *SFRP2* had a favorable prognosis.

## Supplementary information

**Additional file 1: Table S1.** Primers and conditions for MS-HRM analysis.

**Additional file 2: Figure S1.** The genome position of the amplicon in this study and that of cg probe in the TCGA.

**Additional file 3: Table S2.** The range and comparison of gene methylation levels in tumor tissues and adjacent non-tumor tissues.

**Additional file 4: Table S3.** Comparisons of survival time between groups stratified by methylation levels of genes.

**Additional file 5: Table S4.** Univariate and multivariate Cox analysis for association between variables and OS in 307 CRC patients.

**Additional file 6: Table S5.** Univariate and multivariate Cox analysis for association between clinic characteristics, methylation and OS in TCGA.

**Additional file 7: Table S6.** Univariate and multivariate Cox analysis for association of cg methylation of *SFRP1*, *SFRP2*, and *WIF1* with OS in TCGA.

## Data Availability

The datasets used and/or analysed during the current study are available from the corresponding author on reasonable request.

## Abbreviations

AUC: Area under curve
CI: Confidence intervals
CRC: Colorectal cancer
HR: Hazard ratio
MS-HRM: Methylation-sensitive high resolution melting
OS: Overall survival
PS: Propensity score
ROC: Receiver operator characteristic
*SFRP1*: Secreted frizzled-related protein 1
*SFRP2*: Secreted frizzled-related protein 2
*SFRPs*: Secreted frizzled-related proteins
TCGA: The Cancer Genome Atlas
*WIF1*: Wnt inhibitory factor 1
