# Supplementary material for: DNA methylation of SFRP1, SFRP2, and WIF1 and prognosis of postoperative colorectal cancer patients
Source: BMC Cancer. 2019 Dec 12;19:1212. doi: 10.1186/s12885-019-6436-0 (PMC6909551; doi:10.1186/s12885-019-6436-0)
Supplement: Supplementary file 1 — Additional file 1: Table S1. Primers and conditions for MS-HRM analysis. [file 12885_2019_6436_MOESM1_ESM.docx]

**Additional file 1**

**Table S1 Primers and conditions for MS-HRM analysis**

| **Gene** | **Primer sequences 5’-3’** | **CpG sites/**  **amplicon length** | **Annealing temperature(°C)** |
| --- | --- | --- | --- |
| *SFRP1* | F: GGAAAGAGATAAGGGGAGAAAAAGAA | 7/139 | 60-56 |
|  | R: ATTTCATAAATTTACAAATATAATCCAAACTCC |  |  |
| *SFRP2* | F:GGTTAAGATAGGTTTAATTGATTATTGGGGAATAG | 13/119 | 60-56 |
|  | R: TAAACACCCAATATCCCATCCCT |  |  |
| *WIF1* | F:GAGGTGGCGAGTGATGTTTTAGG | 10/121 | 62-58 |
|  | R:AACCCCCGAAACTACATTCACAATA |  |  |
