# Supplementary material for: DNA methylation of SFRP1, SFRP2, and WIF1 and prognosis of postoperative colorectal cancer patients
Source: BMC Cancer. 2019 Dec 12;19:1212. doi: 10.1186/s12885-019-6436-0 (PMC6909551; doi:10.1186/s12885-019-6436-0)
Supplement: Supplementary file 3 — Additional file 3: Table S2. The range and comparison of gene methylation levels in tumor tissues and adjacent non-tumor tissues. [file 12885_2019_6436_MOESM3_ESM.docx]

**Additional file 3**

**Table S2 The range and comparison of gene methylation levels in tumor tissues and adjacent non-tumor tissues.**

| **Gene** | **Methylation levels** | | ***P*^a^** |
| --- | --- | --- | --- |
|  | **Tumor tissues** | **Non-tumor tissues** |  |
| *SFRP1* | 2.5-60.0% | 0.0-15.0% | < 0.001 |
| *SFRP2* | 1.0-60.0% | 0.0-20.0% | < 0.001 |
| *WIF1* | 2.5-60.0% | 15.0-35.0% | < 0.001 |

^a^ Mann-Whitney U test
