# Supplementary material for: DNA methylation of SFRP1, SFRP2, and WIF1 and prognosis of postoperative colorectal cancer patients
Source: BMC Cancer. 2019 Dec 12;19:1212. doi: 10.1186/s12885-019-6436-0 (PMC6909551; doi:10.1186/s12885-019-6436-0)
Supplement: Supplementary file 4 — Additional file 4: Table S3. Comparisons of survival time between groups stratified by methylation levels of genes. [file 12885_2019_6436_MOESM4_ESM.docx]

**Additional file 4**

**Table S3 Comparisons of survival time between groups stratified by methylation levels of genes**

| **Gene** | **Methylation levels** | **OS (months)** | | ***P*** |
| --- | --- | --- | --- | --- |
|  |  | **Mean (SE) ^a^** | **Median** |  |
| All subjects |  | 76.90 (2.26) | 73 |  |
| *SFRP1* | Hypomethlation | 60.71 (5.32) | 52 | 0.001 |
|  | Hypermethlation | 79.98 (2.42) | >102 |  |
| *SFRP2* | Hypomethlation | 73.37 (2.48) | 83 | 0.000 |
|  | Hypermethlation | 96.96 (4.29) | >102 |  |
| *WIF1* | Hypomethlation | 76.12 (2.58) | 96 | 0.377 |
|  | Hypermethlation | 79.29 (4.56) | >102 |  |
| Co-methylation-2 | Co-methylation-2L | 73.37 (2.48) | 83 | 0.000 |
|  | Co-methylation-2H | 96.96 (4.29) | >102 |  |
| Co-methylation-3 | Co-methylation-3L | 75.13 (2.37) | 95 | 0.010 |
|  | Co-methylation-3H | 97.14 (6.05) | >102 |  |

^a^ Mean survival time (standard error)
