# Supplementary material for: DNA methylation of SFRP1, SFRP2, and WIF1 and prognosis of postoperative colorectal cancer patients
Source: BMC Cancer. 2019 Dec 12;19:1212. doi: 10.1186/s12885-019-6436-0 (PMC6909551; doi:10.1186/s12885-019-6436-0)
Supplement: Supplementary file 5 — Additional file 5: Table S4. Univariate and multivariate Cox analysis for association between variables and OS in 307 CRC patients. [file 12885_2019_6436_MOESM5_ESM.docx]

**Additional file 5**

**Table S4 Univariate and multivariate Cox analysis for association between variables and OS in 307 CRC patients**

| **Variables** | **Number** | | **Univariate Cox** | | **Multivariate Cox** | |
| --- | --- | --- | --- | --- | --- | --- |
|  | **Patients (N = 307)** | **Deaths (N = 127)** | **Crude HR (95%CI)** | ***P*** | **Adjusted HR (95%CI)** | ***P*** |
| Age |  |  |  | -- ^a^ |  | -- ^a^ |
| < 45 years-old | 33 | 16 | 1.000 |  | 1.000 |  |
| 45~60 years-old | 132 | 49 | 0.784 (0.445-1.378) |  | 0.508 (0.282-0.916) ^b^ |  |
| ≥ 60 years-old | 142 | 62 | 0.942 (0.543-1.632) |  | 0.879 (0.500-1.544) |  |
| Gender |  |  |  | 0.462 |  | 0.143 |
| Male | 180 | 72 | 1.000 |  | 1.000 |  |
| Female | 127 | 55 | 1.141 (0.803-1.621) |  | 1.225 (0.848-1.770) |  |
| CEA |  |  |  | **0.001** |  | 0.976 |
| < 5 ng/mL | 130 | 39 | 1.000 |  | 1.000 |  |
| ≥ 5 ng/mL | 177 | 88 | 1.860 (1.275-2.713) |  | 1.318 (0.911-1.908) |  |
| CA19-9 |  |  |  | **0.000** |  | **0.000** |
| < 37 U/mL | 232 | 63 | 1.000 |  | 1.000 |  |
| ≥ 37 U/mL | 75 | 64 | 5.151 (3.609-7.353) |  | 4.208 (2.756-6.424) |  |
| Multiple polyps |  |  |  | 0.416 |  | -- |
| No | 220 | 94 | 1.000 |  | -- |  |
| Yes | 87 | 33 | 0.824 (0.514-1.323) |  | -- |  |
| Tumor location |  |  |  | 0.474 |  | -- |
| Colon | 115 | 44 | 1.000 |  | -- |  |
| Rectum | 192 | 83 | 1.143 (0.793-1.647) |  | -- |  |
| TNM Staging |  |  |  | **0.000** |  | **0.000** |
| I- II | 163 | 42 | 1.000 |  | 1.000 |  |
| III-IV | 144 | 85 | 3.176 (2.191-4.604) |  | 2.944 (1.999-4.337) |  |
| Pathological classification |  |  |  | -- ^a^ |  | -- ^a^ |
| Prominence | 199 | 64 | 1.000 |  | 1.000 |  |
| Ulceration | 86 | 48 | 1.943 (1.336-2.827) ^b^ |  | 1.320 (0.892-1.953) |  |
| Others | 22 | 15 | 3.597 (2.047-6.321) ^b^ |  | 2.631 (1.440-4.807) ^b^ |  |
| Histologic classification |  |  |  | -- ^a^ |  | -- |
| Adenocarcinoma | 235 | 98 | 1.000 |  | -- |  |
| Mucinous adenocarcinoma | 68 | 28 | 1.066 (0.700-1.624) |  | -- |  |
| Others | 4 | 1 | 0.451 (0.063-3.236) |  | -- |  |
| Differentiation degree |  |  |  | **0.010** |  | **0.023** |
| Poor | 50 | 26 | 1.000 |  | 1.000 |  |
| Moderate or well | 257 | 101 | 0.560 (0.359-0.872) |  | 0.577 (0.360-0.926) |  |
| Postoperative chemotherapy |  |  |  | 0.747 |  | -- |
| Yes | 169 | 69 | 1.000 |  | -- |  |
| No | 138 | 58 | 0.944 (0.666-1.339) |  | -- |  |
| Postoperative radiotherapy |  |  |  | **0.000** |  | **0.010** |
| Yes | 290 | 113 | 1.000 |  | 1.000 |  |
| No | 17 | 14 | 3.085 (1.762-5.404) |  | 2.208 (1.204-4.048) |  |

^a^ “Pooled” data was not provided by SPSS 23.0;

^b^ *P* < 0.05
