# Supplementary material for: DNA methylation of SFRP1, SFRP2, and WIF1 and prognosis of postoperative colorectal cancer patients
Source: BMC Cancer. 2019 Dec 12;19:1212. doi: 10.1186/s12885-019-6436-0 (PMC6909551; doi:10.1186/s12885-019-6436-0)
Supplement: Supplementary file 6 — Additional file 6: Table S5. Univariate and multivariate Cox analysis for association between clinic characteristics, methylation and OS in TCGA. [file 12885_2019_6436_MOESM6_ESM.docx]

**Additional file 6**

**Table S5 Univariate and multivariate Cox analysis for association between clinic characteristics, methylation and OS in TCGA**

| **Variables** | **Number** | | **Univariate Cox** | | **Multivariate Cox** | |
| --- | --- | --- | --- | --- | --- | --- |
|  | **Patients (N = 399)** | **Deaths (N = 89)** | **Crude HR (95%CI)** | ***P*** | **Adjusted HR (95%CI)** | ***P*** |
| Age |  |  |  | 0.627 |  | 0.719 |
| < 45 years-old | 31 | 6 | 1.000 |  | 1.000 |  |
| ≥ 45 years-old | 368 | 83 | 0.814 (0.354-1.869) |  | 1.171 (0.496-2.763) |  |
| Gender |  |  |  | 0.195 |  | 0.237 |
| Male | 215 | 52 | 1.000 |  | 1.000 |  |
| Female | 184 | 37 | 0.756 (0.495-1.154) |  | 0.767 (0.493-1.192) |  |
| CEA |  |  |  | **0.027** |  | **0.043** |
| < 5 ng/mL | 244 | 44 | 1.000 |  | 1.000 |  |
| ≥ 5 ng/mL | 155 | 45 | 2.145 (1.108-4.151) |  | 1.958 (1.026-3.739) |  |
| Multiple polyps |  |  |  | 0.717 |  | -- |
| No | 302 | 71 | 1.000 |  | -- |  |
| Yes | 97 | 18 | 0.880 (0.427-1.815) |  | -- |  |
| Tumor location |  |  |  | 0.583 |  | -- |
| Colon | 303 | 71 | 1.000 |  | -- |  |
| Rectum | 96 | 18 | 0.865 (0.514-1.453) |  | -- |  |
| TNM Staging |  |  |  | **0.001** |  | **0.004** |
| I- II | 214 | 32 | 1.000 |  | 1.000 |  |
| III-IV | 185 | 57 | 2.527 (1.515-4.216) |  | 2.283 (1.331-3.918) |  |
| Histologic classification |  |  |  | 0.782 |  | -- |
| Adenocarcinoma | 350 | 79 | 1.000 |  | -- |  |
| Mucinous adenocarcinoma | 49 | 10 | 1.098 (0.568-2.123) |  | -- |  |
| *SFRP1* (*cg04255616*) |  |  |  | 0.180 |  | 0.593 |
| Hypomethylation | 238 | 56 | 1.000 |  | 1.000 |  |
| Hypermethylation | 161 | 33 | 0.744 (0.484-1.146) |  | 0.886 (0.567-1.384) |  |
| *SFRP2* (*cg25185173*) |  |  |  | 0.756 |  | 0.605 |
| Hypomethylation | 132 | 30 | 1.000 |  | 1.000 |  |
| Hypermethylation | 267 | 59 | 1.072 (0.690-1.665) |  | 1.129 (0.713-1.789) |  |
| *WIF1* |  |  |  | **0.002** |  | **0.002** |
| Hypomethylation | 243 | 44 | 1.000 |  | 1.000 |  |
| Hypermethylation | 156 | 45 | 1.925 (1.269-2.920) |  | 2.022 (1.309-3.124) |  |
| Co-methylation-2 |  |  |  | 0.564 |  | 0.762 |
| Co-methylation-2L ^a^ | 281 | 64 | 1.000 |  | 1.000 |  |
| Co-methylation-2H ^b^ | 118 | 25 | 0.872 (0.549-1.386) |  | 1.078 (0.664-1.747) |  |
| Co-methylation-3 |  |  |  | 0.848 |  | 0.362 |
| Co-methylation-3L ^c^ | 316 | 72 | 1.000 |  | 1.000 |  |
| Co-methylation-3H ^d^ | 83 | 17 | 1.053 (0.620-1.789) |  | 1.297 (0.741-2.271) |  |

^a^ Co-methylation-2L: patients with promoter hypomethylation of at least one gene (*SFRP1* or *SFRP2*)

^b^ Co-methylation-2H: patients with promoter hypermethylation of *SFRP1* and *SFRP2*

^c^ Co-methylation-3L: patients with promoter hypomethylation of at least one gene (*SFRP1* or *SFRP2* or *WIF1*)

^d^ Co-methylation-3H: patients with promoter hypermethylation of *SFRP1*, *SFRP2* and *WIF1*
