# Supplementary material for: DNA methylation of SFRP1, SFRP2, and WIF1 and prognosis of postoperative colorectal cancer patients
Source: BMC Cancer. 2019 Dec 12;19:1212. doi: 10.1186/s12885-019-6436-0 (PMC6909551; doi:10.1186/s12885-019-6436-0)
Supplement: Supplementary file 7 — Additional file 7: Table S6. Univariate and multivariate Cox analysis for association of cg methylation of SFRP1, SFRP2, and WIF1 with OS in TCGA. [file 12885_2019_6436_MOESM7_ESM.docx]

**Additional file 7**

**Table S6 Univariate and multivariate Cox analysis for association of cg methylation of *SFRP1*, *SFRP2*, and *WIF1* with OS in TCGA**

|  | **Univariate Cox** | | **Multivariate Cox** | |
| --- | --- | --- | --- | --- |
|  | **Crude HR (95%CI)** | ***P*** | **Adjusted HR (95%CI)** | ***P*** |
| *SFRP1* |  |  |  |  |
| *cg00000321* | 1.265(0.786-2.036) | 0.332 | 1.492(0.892-2.495) | 0.127 |
| *cg00930833* | 1.108(0.689-1.783) | 0.671 | 1.028(0.631-1.673) | 0.912 |
| *cg01074584* | 0.782(0.415-1.472) | 0.446 | 0.818(0.427-1.569) | 0.546 |
| *cg01495122* | 0.752(0.491-1.152) | 0.190 | 0.805(0.523-1.239) | 0.323 |
| *cg02154585* | 1.026(0.495-2.123) | 0.946 | 1.036(0.495-2.167) | 0.926 |
| *cg02388150* | 1.015(0.670-1.539) | 0.944 | 1.075(0.702-1.644) | 0.740 |
| *cg03133371* | 0.688(0.317-1.490) | 0.343 | 0.807(0.361-1.806) | 0.602 |
| *cg03575666* | 1.154(0.722-1.844) | 0.550 | 1.101(0.681-1.779) | 0.694 |
| ***cg04255616*** | **0.744(0.484-1.146)** | **0.180** | **0.886(0.567-1.384)** | **0.593** |
| *cg06166767* | 0.922(0.606-1.401) | 0.703 | 1.010(0.659-1.549) | 0.964 |
| *cg06777844* | 1.379(0.880-2.162) | 0.161 | 1.455(0.918-2.306) | 0.110 |
| *cg07122178* | 0.757(0.486-1.181) | 0.220 | 0.763(0.487-1.195) | 0.237 |
| *cg07296835* | 0.931(0.548-1.582) | 0.792 | 0.967(0.559-1.673) | 0.905 |
| *cg07935886* | 1.405(0.884-2.231) | 0.150 | 1.530(0.959-2.443) | 0.075 |
| *cg09410389* | 1.380(0.831-2.293) | 0.214 | 1.378(0.820-2.318) | 0.226 |
| *cg10406295* | 1.335(0.871-2.046) | 0.185 | 1.525(0.988-2.353) | 0.057 |
| *cg13154925* | 1.013(0.668-1.536) | 0.952 | 0.961(0.624-1.479) | 0.856 |
| *cg13398291* | 1.007(0.652-1.556) | 0.976 | 1.160(0.737-1.827) | 0.521 |
| *cg14548509* | 1.101(0.584-2.073) | 0.766 | 1.031(0.537-1.978) | 0.928 |
| *cg14824386* | 1.250(0.736-2.123) | 0.408 | 1.348(0.779-2.332) | 0.286 |
| *cg14904908* | 0.978(0.643-1.488) | 0.918 | 1.055(0.686-1.621) | 0.808 |
| *cg15839448* | 0.912(0.599-1.387) | 0.665 | 1.000(0.654-1.531) | 0.998 |
| *cg16498741* | 1.068(0.676-1.687) | 0.779 | 1.112(0.697-1.775) | 0.656 |
| *cg16662821* | 1.088(0.676-1.751) | 0.729 | 1.244(0.746-2.075) | 0.401 |
| *cg16667459* | 1.091(0.720-1.654) | 0.682 | 1.210(0.789-1.856) | 0.383 |
| *cg17486234* | 0.852(0.488-1.489) | 0.574 | 0.730(0.410-1.299) | 0.285 |
| *cg17816908* | 0.958(0.593-1.547) | 0.860 | 1.005(0.617-1.638) | 0.982 |
| *cg21517947* | 0.856(0.536-1.368) | 0.516 | 0.879(0.540-1.431) | 0.604 |
| *cg21846232* | 1.235(0.781-1.952) | 0.366 | 1.313(0.818-2.110) | 0.259 |
| *cg22418909* | 1.060(0.678-1.656) | 0.799 | 1.184(0.750-1.870) | 0.469 |
| *cg23331238* | 1.284(0.820-2.011) | 0.275 | 1.185(0.749-1.874) | 0.469 |
| *cg23359714* | 0.968(0.630-1.487) | 0.883 | 0.987(0.641-1.522) | 0.954 |
| *cg24319902* | 0.772(0.495-1.205) | 0.255 | 0.739(0.469-1.165) | 0.192 |
| *cg25927227* | 0.785(0.480-1.284) | 0.335 | 0.709(0.431-1.166) | 0.176 |
| *ch.8.969355F* | 0.678(0.445-1.033) | 0.070 | 0.791(0.508-1.231) | 0.299 |
| *allcgofSFRP1^a^* | 1.139(0.653-1.986) | 0.646 | 1.250(0.710-2.202) | 0.439 |
| *SFRP2* |  |  |  |  |
| *cg00082664* | 1.322(0.846-2.065) | 0.220 | 1.397(0.880-2.217) | 0.156 |
| *cg00705808* | 0.833(0.542-1.282) | 0.406 | 0.863(0.552-1.350) | 0.520 |
| *cg01298731* | 1.032(0.658-1.616) | 0.892 | 1.001(0.637-1.572) | 0.997 |
| *cg03202804* | 1.188(0.774-1.824) | 0.430 | 1.228(0.789-1.910) | 0.364 |
| *cg04965141* | 1.193(0.768-1.851) | 0.432 | 1.341(0.850-2.115) | 0.207 |
| *cg05050042* | 0.876(0.560-1.371) | 0.563 | 0.880(0.561-1.381) | 0.580 |
| *cg05164933* | 1.156(0.759-1.761) | 0.500 | 1.308(0.850-2.013) | 0.222 |
| *cg05241277* | 1.006(0.662-1.528) | 0.979 | 0.987(0.648-1.504) | 0.951 |
| *cg05774801* | 1.454(0.875-2.415) | 0.149 | 1.462(0.862-2.481) | 0.159 |
| *cg05874561* | 1.097(0.719-1.674) | 0.668 | 1.216(0.785-1.885) | 0.381 |
| *cg06549216* | 1.494(0.773-2.890) | 0.233 | 1.740(0.884-3.425) | 0.109 |
| *cg07694025* | 1.301(0.783-2.164) | 0.310 | 1.363(0.808-2.302) | 0.246 |
| *cg07859799* | 0.917(0.590-1.427) | 0.702 | 1.087(0.678-1.745) | 0.728 |
| *cg07999845* | 1.088(0.713-1.659) | 0.695 | 1.104(0.719-1.695) | 0.651 |
| *cg09788843* | 0.769(0.500-1.184) | 0.234 | 0.916(0.576-1.457) | 0.710 |
| *cg10318528* | 0.965(0.608-1.532) | 0.881 | 0.874(0.540-1.414) | 0.582 |
| *cg10663078* | 0.959(0.630-1.460) | 0.844 | 0.913(0.598-1.395) | 0.675 |
| *cg10790791* | 0.809(0.391-1.675) | 0.568 | 0.965(0.455-2.045) | 0.926 |
| *cg10942078* | 1.154(0.760-1.754) | 0.501 | 1.342(0.875-2.057) | 0.177 |
| *cg11354906* | 1.550(0.839-2.863) | 0.162 | 1.446(0.769-2.721) | 0.253 |
| *cg11467638* | 0.834(0.478-1.454) | 0.522 | 0.848(0.485-1.481) | 0.561 |
| *cg13357229* | 0.854(0.554-1.318) | 0.477 | 0.994(0.630-1.568) | 0.978 |
| *cg13732865* | 0.865(0.569-1.316) | 0.498 | 0.839(0.549-1.281) | 0.416 |
| *cg14063488* | 1.262(0.767-2.078) | 0.360 | 1.481(0.886-2.478) | 0.134 |
| *cg14289246* | 1.113(0.617-2.007) | 0.722 | 1.180(0.633-2.198) | 0.603 |
| *cg14330641* | 0.958(0.631-1.456) | 0.841 | 1.025(0.666-1.580) | 0.910 |
| *cg14435644* | 0.749(0.479-1.173) | 0.207 | 0.821(0.517-1.303) | 0.403 |
| *cg20727217* | 0.837(0.479-1.461) | 0.531 | 1.009(0.562-1.812) | 0.976 |
| *cg20881942* | 1.110(0.699-1.765) | 0.658 | 1.064(0.662-1.710) | 0.798 |
| *cg21657059* | 0.961(0.578-1.598) | 0.878 | 1.030(0.605-1.752) | 0.914 |
| *cg22178613* | 1.109(0.535-2.300) | 0.780 | 1.037(0.459-2.346) | 0.929 |
| *cg23121156* | 1.391(0.858-2.253) | 0.180 | 1.430(0.859-2.381) | 0.169 |
| *cg23207990* | 1.314(0.698-2.472) | 0.398 | 1.157(0.564-2.373) | 0.687 |
| *cg23292160* | 1.141(0.726-1.795) | 0.567 | 1.214(0.758-1.946) | 0.420 |
| *cg23502475* | 0.727(0.472-1.119) | 0.147 | 0.807(0.513-1.269) | 0.352 |
| *cg23714408* | 1.298(0.843-1.998) | 0.236 | 1.311(0.841-2.044) | 0.232 |
| *cg23910835* | 1.040(0.685-1.580) | 0.853 | 1.263(0.791-2.018) | 0.326 |
| *cg24241928* | 0.796(0.516-1.229) | 0.303 | 0.895(0.571-1.404) | 0.630 |
| *cg24372829* | 1.180(0.655-2.126) | 0.582 | 1.222(0.669-2.235) | 0.514 |
| *cg24968721* | 0.783(0.448-1.369) | 0.392 | 0.705(0.399-1.244) | 0.228 |
| *cg25645268* | 1.372(0.889-2.116) | 0.153 | 1.515(0.971-2.365) | 0.067 |
| *cg25775322* | 1.512(0.970-2.357) | 0.068 | 1.552(0.977-2.466) | 0.063 |
| ***cg25185173*** | **1.072(0.690-1.665)** | **0.756** | **1.129(0.713-1.789)** | **0.605** |
| *allcgofSFRP2^a^* | 1.165(0.759-1.788) | 0.485 | 1.336(0.859-2.078) | 0.199 |
| *WIF1* |  |  |  |  |
| *cg03509412* | 1.292(0.844-1.979) | 0.239 | 1.471(0.956-2.264) | 0.079 |
| *cg10065957* | 1.169(0.731-1.870) | 0.514 | 1.032(0.634-1.679) | 0.900 |
| *cg15862358* | 0.940(0.600-1.473) | 0.788 | 0.940(0.596-1.482) | 0.790 |
| *cg16009877* | 1.329(0.858-2.058) | 0.203 | 1.328(0.849-2.078) | 0.213 |
| *cg16099107* | 1.094(0.713-1.678) | 0.680 | 1.027(0.666-1.582) | 0.905 |
| *cg19427610* | 1.440(0.949-2.185) | 0.086 | ***1.546(1.012-2.362)*** | ***0.044*** |
| *cg20098478* | 1.086(0.714-1.651) | 0.701 | 1.124(0.736-1.716) | 0.587 |
| *cg21383810* | 0.934(0.557-1.568) | 0.797 | 0.898(0.530-1.521) | 0.688 |
| *cg24166864* | 1.078(0.654-1.775) | 0.769 | 1.138(0.682-1.898) | 0.622 |
| *cg26397188* | 1.410(0.877-2.269) | 0.157 | 1.510(0.931-2.447) | 0.095 |
| *cg26733786* | 1.475(0.967-2.251) | 0.071 | ***1.597(1.039-2.455)*** | ***0.033*** |
| ***allcgofWIF1****^a^* | **1.925(1.269-2.920)** | **0.002** | **2.022(1.039-3.124)** | **0.002** |

^a^ the average of all probes methylation values of gene
